# Wheat bran promotes enrichment within the human colonic microbiota of butyrate‐producing bacteria that release ferulic acid

**DOI:** 10.1111/1462-2920.13158

**Published:** 2016-01-21

**Authors:** Sylvia H. Duncan, Wendy R. Russell, Andrea Quartieri, Maddalena Rossi, Julian Parkhill, Alan W. Walker, Harry J. Flint

**Affiliations:** ^1^ Rowett Institute of Nutrition and HealthUniversity of AberdeenAberdeenUK; ^2^ Department of Life SciencesUniversity of Modena and Reggio EmiliaModenaItaly; ^3^ Pathogen Genomics GroupWellcome Trust Sanger InstituteHinxtonCambridgeshireUK

## Abstract

Cereal fibres such as wheat bran are considered to offer human health benefits via their impact on the intestinal microbiota. We show here by 16S rRNA gene‐based community analysis that providing amylase‐pretreated wheat bran as the sole added energy source to human intestinal microbial communities in anaerobic fermentors leads to the selective and progressive enrichment of a small number of bacterial species. In particular, OTUs corresponding to uncultured Lachnospiraceae (Firmicutes) related to *E* *ubacterium xylanophilum* and *B* *utyrivibrio* spp. were strongly enriched (by five to 160 fold) over 48 h in four independent experiments performed with different faecal inocula, while nine other Firmicutes OTUs showed > 5‐fold enrichment in at least one experiment. Ferulic acid was released from the wheat bran during degradation but was rapidly converted to phenylpropionic acid derivatives via hydrogenation, demethylation and dehydroxylation to give metabolites that are detected in human faecal samples. Pure culture work using bacterial isolates related to the enriched OTUs, including several butyrate‐producers, demonstrated that the strains caused substrate weight loss and released ferulic acid, but with limited further conversion. We conclude that breakdown of wheat bran involves specialist primary degraders while the conversion of released ferulic acid is likely to involve a multi‐species pathway.

## Introduction

Non‐digestible fibre such as cereal bran is considered to be an important component of a healthy human diet (Nilsson *et al*., 2008; Smith and Tucker, 2011; Flint *et al*., 2012). Fibre can affect health through multiple mechanisms, which include its influence on gut transit, prebiotic effects, its fermentation in the large intestine to yield short‐chain fatty acids (SCFA), and its contribution to the host metabolome of potentially health‐protective compounds of plant origin (Lampe *et al*., 1992; Gill and Rowland, 2002; Dhingra *et al*., 2012). Most of these effects depend on the activities of the resident microbiota within the large intestine, which has the capacity to degrade fibre that cannot be digested by the host's digestive enzymes. Feeding of wheat bran has been shown to decrease chemically induced tumours in rodents, and this effect has been ascribed largely, but not exclusively, to the stimulation of butyrate formation via wheat bran fermentation in the large intestine (McIntyre *et al*., 1993; Zoran *et al*., 1997; Compher *et al*., 1999; Damen *et al*., 2011; Mikkelsen *et al*., 2011; Louis *et al*., 2014).

Microbial degradation of cereal bran also leads to the release of phenolic compounds that are detected in faecal samples (Russell *et al*., 2011), e.g. compounds such as ferulic acid, which are integral components within plant cell wall structures (Bunzel *et al*., 2001; Manach *et al*., 2004). These compounds can be taken up into the circulation and therefore have the potential to influence systemic health in addition to gut health. Phenolic compounds can act through a variety of mechanisms, including as antioxidants, upregulators of host redox systems, as anti‐inflammatory agents and as promoters of apoptosis in cancer cells (Stevenson and Hurst, 2007; Islam *et al*., 2008). These diverse mechanisms are thought to underlie the epidemiological and experimental evidence of protection against cardiovascular disease and cancer from increased fibre consumption (World Cancer Research Fund/American Institute of Cancer Research report, 2007; Louis *et al*., 2014). While some phenolic compounds are derived from the fermentation of aromatic amino acids, plant fibre appears to be the major source of phenolic compounds that are considered to benefit health (Jenner *et al*., 2005; Russell *et al*., 2011; 2013).

There is surprisingly little understanding of the microbial ecology of fibre breakdown in the human colon, particularly for complex, insoluble, forms of fibre such as cereal bran that consist primarily of plant cell walls (Faulds, 2010). Previous studies showed that different bacterial species were promoted in the faecal microbiota of human volunteers fed controlled diets enriched with wheat bran compared with resistant starch (Walker *et al*., 2011; Salonen *et al*., 2014). This agrees with earlier work showing that different bacterial 16S rRNA gene sequences became enriched in incubations with insoluble wheat bran, resistant starch or porcine mucin in an *in vitro* fermentor system, indicating a high degree of substrate specificity among the species colonizing these substrates (Leitch *et al*., 2007). Release of phenolic acids from wheat bran by human gut microbiota has been demonstrated *in vitro* using batch cultures (Nordlund *et al*., 2012).

Our objective here was to analyse the colonization of wheat bran by human colonic microbiota. For this we used the *in vitro* fermentor system described by Leitch and colleagues (2007), in which the insoluble substrate is incubated under conditions of controlled pH and a continuous input of sterile anaerobic medium. We report the sequence of changes in microbiota composition with time during colonization of wheat bran using high‐throughput 16S rRNA gene sequencing and identify for the first time the major species likely to be responsible for wheat bran degradation in the human colon. At the same time, we were able to follow the release and transformation of wheat bran‐derived phenolic metabolites by the associated microbial community.

## Results

### Microbial community succession during growth on wheat bran

The colonization of wheat bran by human colonic bacteria was investigated using a continuous flow anaerobic fermentor system adapted for insoluble substrates (Leitch *et al*., 2007). For each experiment the substrate compartment was inoculated with microbiota from a fresh faecal sample provided by a healthy donor; samples were taken after 2, 4, 8, 24 and 48 h incubation and processed to yield S (solid substrate) and L (liquid) fractions (see *Experimental procedures* section). Four experiments were conducted, each with a different adult faecal donor. In all cases an increase in colonization of the substrate by bacteria, especially by Lachnospiraceae, during incubation was clearly evident from fluorescent *in situ* hybridization (FISH) analysis (Fig. S1). The DNA extracted from each sample was amplified with barcoded primers targeting the 16S rRNA gene, and the resulting amplicons (V3–V5 regions) were then sequenced using 454 pyrosequencing. Detailed analysis at the operational taxonomic unit (OTU) level (97% cut‐off) revealed a total of 406 OTUs, of which 14 accounted individually for > 2% and 23 for > 1% of total sequences. As differences between S and L fractions were shown to be insignificant using metastats, the summed (S + L) data for each time point are considered here.

**Figure 1:**
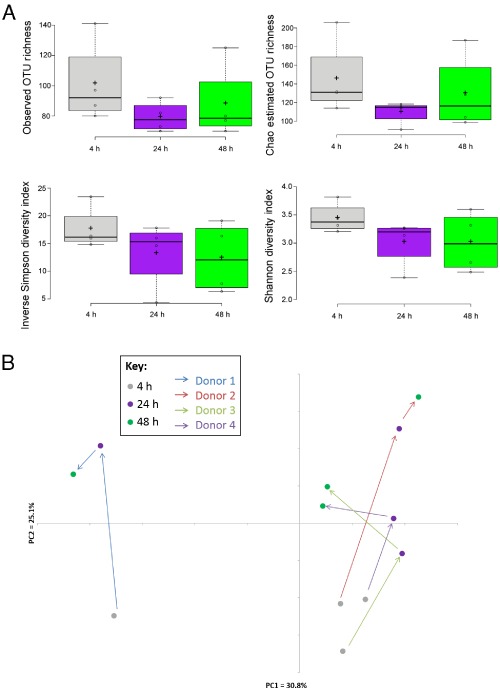
Diversity of microbial communities derived from human faecal inocula with wheat bran as the sole added energy source. A. Four different measures of diversity (observed OTU richness, Chao estimate of total richness, Shannon diversity index and inverse Simpson diversity index) are shown, based on amplified 16S rRNA gene sequences for 4, 24 and 48 h time points. Centre lines show the medians; crosses represent sample means; box limits indicate the 25th and 75th percentiles as determined by R software; whiskers extend 1.5 times the interquartile range from the 25th and 75th percentiles, outliers are represented by dots; data points are plotted as open circles. Plotted using boxplotr. B. PCoA plot showing temporal changes in community composition for each of the four experiments. The coloured arrows indicate the community structure movement over time for each faecal donor. The R‐squared value between the calculated OTU distance matrix and the distance between the points in the two‐dimensional PCoA plot space was 0.83.

**Figure 2:**
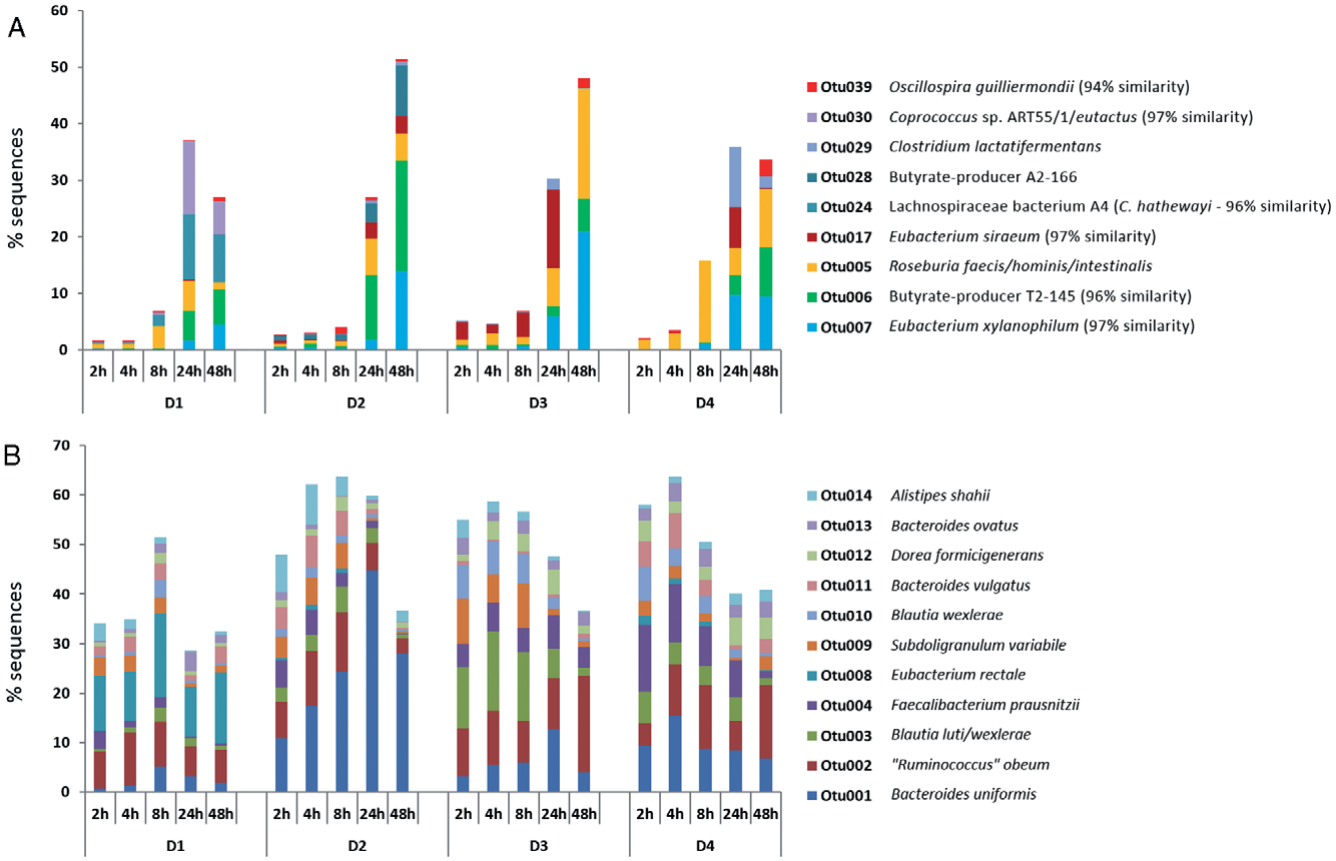
Relative abundance of 16S rRNA gene OTUs in anaerobic fermentor communities provided with wheat bran as sole energy source. D1, D2, D3 and D4 refer to four separate experiments, each inoculated with faecal microbiota from a different healthy individual. A. Nine OTUs that were strongly enriched during growth on wheat bran (> 5‐fold increase in proportional abundance between 4 and 48 h for at least one inoculum). B. Temporal changes in the most abundant OTUs (each comprising > 2% of total sequences) detected across the four experiments (not including the enriched OTUs 5, 6 and 7, already shown in A).

Overall bacterial community diversity, as assessed by calculating observed OTU richness, Chao estimates of total richness, and the Shannon and Simpson diversity indices, was not significantly different between fermentor systems at each sampling time point, with mean Shannon indices of around 3 at 24 and 48 h (Fig. 1A). There was, however, marked donor‐specific variation in microbiota composition between fermentor experiments (Analysis of molecular variance (AMOVA), *P* = 0.006). Incubation with wheat bran also had a significant impact on the temporal community structure within the fermentors (AMOVA, *P* = 0.041), and the impacts of both inter‐donor variation and incubation with wheat bran on microbiota composition were visualized using principal coordinates analysis (PCoA) (Fig. 1B). More specifically, nine OTUs (all Firmicutes) were identified as becoming strongly enriched (defined as >5‐fold increase in proportional abundance) among amplified 16S rRNA gene sequences at the 24 or 48 h time points relative to the 4 h time point (Fig. 2A, Fig. S2). Across the four experiments these nine OTUs, taken together, accounted for 1.5–5.5% of all sequences at 2 h and 4 h (means: 1.9% and 3.2%, respectively), 4–16% (mean: 8.3%) at 8 h, 27–36% (mean: 32.6%) at 24 h, and 27–52% (mean: 40%) at 48 h. In contrast, 11 of the 14 most abundant OTUs that individually accounted for > 2% total sequences showed little evidence of enrichment, although the proportional abundance of *Bacteroides uniformis* (OTU1) increased approximately fourfold between 4 and 24 h in the experiment inoculated with faeces from D2 (Fig. 2B).

**Figure 3:**
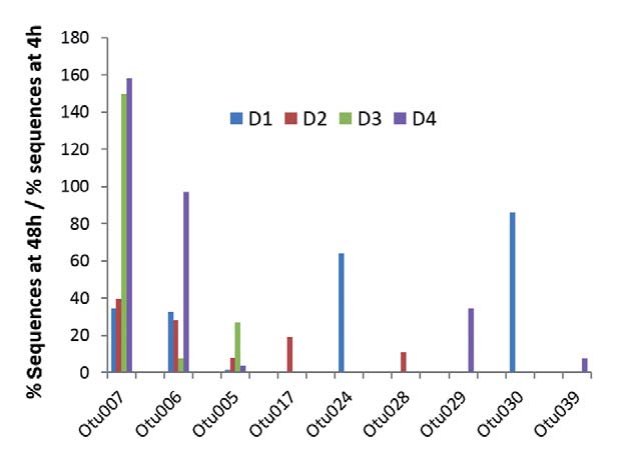
Fold increases in OTU abundance. The ratio of proportional abundance at 48 h to proportional abundance at 4 h (i.e. the fold increase) is shown here for the nine OTUs that were highly enriched during growth on wheat bran (taxonomic classifications for each OTU are shown in Fig. 2A). In addition (not shown) OTU68 (*C* *lostridium xylanolyticum*) increased from a single sequence at the 4 h time point to 80 sequences at 48 h across the four experiments (a significant increase based on LEfSe analysis, Table S2).

Remarkably, three OTUs (all derived from the Lachnospiraceae family) showed enrichment in all four experiments (corresponding to faecal microbiota used as inocula from four different donors). In particular, OTU7 (related to *Eubacterium xylanophilum*) showed a 25‐, 40‐, 150‐ and 160‐fold enrichment, respectively, in the four experiments, while OTU6 (related to the unclassified butyrate‐producing isolate T2‐145, closest species *Butyrivibrio crossotus* – Barcenilla *et al*., 2000) showed 35‐, 30‐, 5‐ and 100‐fold enrichment (Fig. 3). Increases > 5‐fold were also detected in two experiments for OTU5 (corresponding to *Roseburia intestinalis/faecis/hominis*). LEfSe analysis confirmed that these three OTUs were significantly greater at the 48 h relative to the 4 h time point, and also suggested significant enrichment for the less abundant OTU32 (*Clostridium cellulovorans*) and OTU68 (*Clostridium xylanolyticum*) (Table S1). In addition, *Eubacterium siraeum* (Ruminococcaceae) showed enrichment at 24 h in three of the four donors, although in two cases its representation decreased at 48 h. Five additional OTUs shown in Fig. 3 gave individual‐specific enrichments, but had low or undetectable numbers in fermentors inoculated with faeces from all other donors.

### Products of wheat bran fermentation

**Figure 4:**
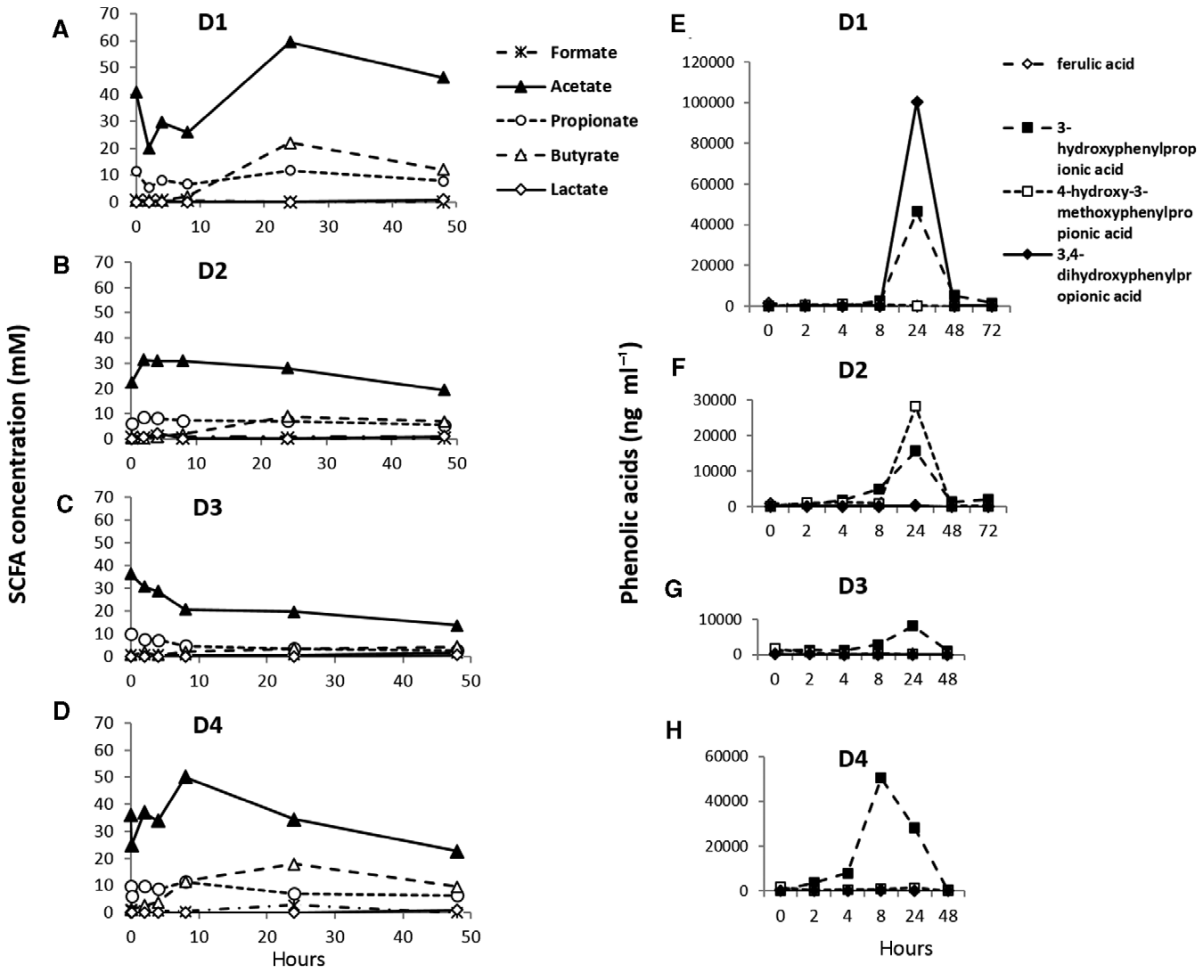
Metabolites arising from fermentation of wheat bran. (A–D) Concentrations (mM) of short‐chain fatty acids. An SCFA mix was added initially to the fermentor vessel medium (see *Experimental procedures*) but is progressively diluted through turnover so that concentrations at 24 and 48h mainly reflect microbial fermentation (see text). (E–H) Concentrations (ng ml^−1^) of ferulic acid and its PPA (phenylpropionic acid) metabolites in anaerobic continuous flow fermentor incubations of pretreated wheat bran with mixed faecal microbiota from four human volunteers (D1‐4). Data refer to the experiments shown in 1, 2, 3.

The production of SCFAs from wheat bran fermentation was monitored in these experiments as a measure of microbial activity (Fig. 4A–D). For the liquid phase within the nylon bags, the mean SCFA proportions due to fermentation for the four experiments (after allowing for residual SCFAs from the initial medium) were acetate 56.5%, propionate 9.2%, butyrate 32.3% at 24 h, and at 48 h, acetate 55.4%, propionate 13.8%, and butyrate 24.3%. Total SCFA concentrations varied considerably between the four experiments, with the D1 inoculum producing the highest, and the D3 inoculum the lowest, rate of fermentation between 0 h and 48 h. As a control, batch cultures were examined with the same medium and received the same faecal inocula, but with no added wheat bran. These cultures yielded (means) 3.31 mM acetate, 1.94 mM propionate and 1.48 mM butyrate in 24 h and 9.23 mM acetate, and 2.46 mM propionate and 1.66 mM butyrate in 48 h, showing that in the fermentor system bacterial enrichment and SCFA production were largely derived from fermentation of the wheat bran rather than the small amount of peptide (0.2%) present in the medium.

Ferulic acid (4OH3OMeCA) accounts for more than half of bound phenolic compounds present in wheat bran (Iiyama *et al*., 1994; Neacsu *et al*., 2013). Concentrations of ferulic acid‐derived phenolic acids (Fig. S3) detected in fermentor liquid from within the nylon bag peaked at 24 h (Fig. 4E–H). Ferulic acid itself was detected only at low concentrations in fermentor supernatant (L samples), which can be explained by rapid hydrogenation to produce substituted phenylpropionic acids (PPA) after release from the wheat bran. For fermentors inoculated with faeces from D3 and D4, the predominant ferulic acid‐derived metabolite was 3‐hydroxyphenylpropionic acid (3‐OHPPA), resulting from demethylation of the methoxyl group at C3 and dehydroxylation at C4. Concentrations of 3‐OHPPA were much lower for D3, consistent with a lower rate of wheat bran degradation as revealed by the changes in SCFA (Fig. 4). For D1, the dominant product was 3,4‐dihydroxyphenylpropionic acid (3,4‐diOHPPA), followed by 3‐OHPPA, which indicates demethylation but retention of the hydroxyl group at C4. For D2, the dominant product at 24 h was 4‐hydroxy‐3‐methoxyphenylpropionic acid (4‐OH,3‐OMePPA), with 3‐methoxyphenylpropionic acid (3‐OMePPA) dominant at 48 h and 72 h, suggesting that demethylation activity was low relative to dehydroxylation.

### Action of isolated human colonic bacteria on wheat bran

**Figure 5:**
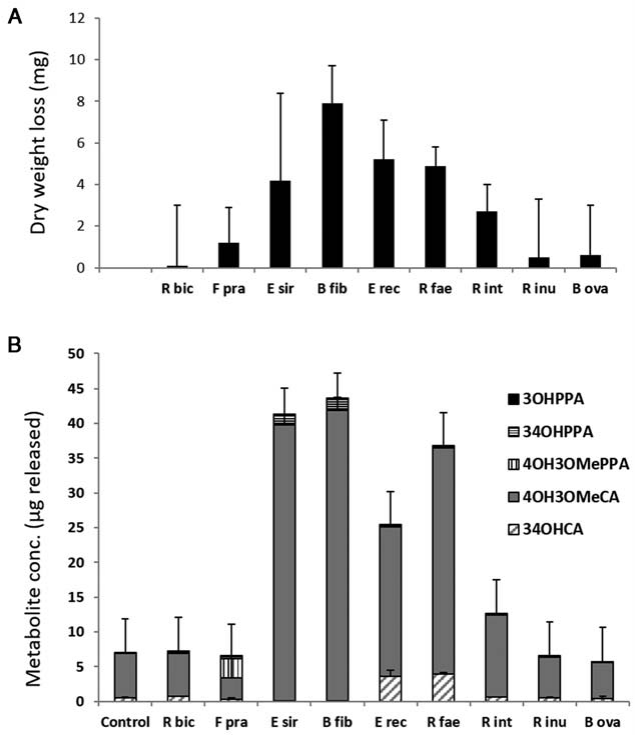
Dry weight loss (A) and release of ferulic acid (B) from wheat bran following incubation with isolated strains of human colonic bacteria. Dry weight losses (shown in A) are from pancreatin‐pretreated wheat bran over 7‐day incubation and represent the means of three separate experiments (each involving triplicate cultures). Released phenolic acids (shown in B) represent means for triplicate cultures after 72 h from a separate experiment involving non‐pretreated wheat bran; phenolic acid release from pretreated wheat bran was similar (not shown). 3,4OHCA is 3,4 dihydroxycinnamic acid; structures of ferulic acid (3OH4OMeCA) and its PPA derivatives are shown in Fig. S3. The following strains were tested: R bic (*R* *uminococcus bicirculans* 80/3); E sir (*E* *ubacterium siraeum* V10sc8a); B fib (*B* *utyrivibrio fibrisolvens* 16/4); R fae (*R* *oseburia faecis* M72/1); E rec (*E* *ubacterium rectale* A1‐86); F pra (*F* *aecalibacterium prausnitzii* A2‐165); B ova (*B* *acteroides ovatus* V975); R int (*R* *oseburia intestinalis* L1‐82); R inu (*R* *oseburia inulinivorans* A2‐194). The following strains fermented the pretreated wheat bran substrate mainly to butyrate: *B* *. fibrisolvens* (8.2 mM butyrate after 7 days); *R* *. intestinalis* (7.8 mM); *R* *. faecis* (6.3 mM) and *E* *. rectale* (4.5 mM) (concentrations from the experiment shown in A, means of triplicate cultures).

The ability of nine isolated human colonic bacteria to degrade wheat bran was also examined in pure culture. Species were chosen for their ability to degrade xylans (*Bacteroides ovatus*, *Eubacterium siraeum*, *Roseburia intestinalis*, *Roseburia faecis*, *Butyrivibrio fibrisolvens*), other hemicelluloses (*Ruminococcus bicirculans*, *Eubacterium rectale*, *Roseburia inulinivorans*) or pectin (*B. ovatus*, *Faecalibacterium prausnitzii*). The greatest loss in substrate dry weight after 7 days was seen with the Lachnospiraceae isolates *B. fibrisolvens*, *E. rectale*, *R. faecis* and *R. intestinalis*, and with the Ruminococcaceae isolate *E. siraeum* (Fig. 5A). These bacteria are among the closest cultured relatives of four OTUs (numbers 5, 6, 7 and 17) that were shown to be enriched in the fermentor experiments at 24 and 48 h using the 16S rRNA gene surveying approach (Fig. 2). Four of these five isolates (all except *E. siraeum*) produced butyrate as their main fermentation product after 7‐day incubation on wheat bran (Fig. 5, legend). These five bacteria were also found to release ferulic acid, further confirming that release of the phenolic acid depends on degradation of the wheat bran. Further transformation (hydrogenation, dehydroxylation, demethylation) of released ferulic acid by these bacterial isolates was, however, very limited (Fig. 5B).

## Discussion

As a dietary component, wheat bran is an insoluble fibre that is non‐digestible in the human upper digestive tract but is subject to bacterial fermentation in the large intestine. We set out here to establish whether certain species of bacteria within the intestinal microbiota are particularly associated with wheat bran fermentation, or conversely whether this substrate favours a wide range of species. The results from our *in vitro* fermentor experiments, using inocula from four healthy individuals, indicate that a small number of species are likely to play key roles in wheat bran fermentation. Thus sequences related to *Eubacterium xylanophilum* became strongly enriched in all four experiments after 48 h incubation. Although it has not been isolated from the human gastrointestinal tract, rumen isolates of *E. xylanophilum* have been reported to ferment xylan to form formate, acetate and butyrate (Van Gylswyck and van der Toorn, 1985). In the present work, two other groups of Lachnospiraceae, related to *Butyrivibrio* spp. and to *Roseburia* spp., also showed enrichment from two or more of the four faecal inocula. This agrees well with findings from an *in vivo* human volunteer study (Salonen *et al*., 2014) in which 16S rRNA gene signals for Lachnospiraceae from faecal samples showed a positive response to a controlled diet that had been enriched with wheat bran. The observation that the relative populations of these species increase within the community does not prove that they are responsible for initiating degradation of the substrate. However, this possibility is strongly suggested by the finding in the present study that cultured relatives of the stimulated OTUs caused weight loss from wheat bran *in vitro*. *Butyrivibrio fibrisolvens* 16/4 is xylanolytic (Rumney *et al*., 1995) and the genus *Roseburia* includes xylanolytic species (Duncan *et al*., 2002; 2006; Chassard *et al*., 2007). Nevertheless, our analysis suggests that several of the most highly stimulated 16S rRNA gene sequences represent uncultured strains or species whose wheat bran‐degrading activity could well be higher than that of the available cultured isolates. Interestingly, the OTUs most highly enriched on wheat bran (most closely similar to *E. xylanophilum*, *Roseburia* spp., *Butyrivibrio fibrisolvens*) are related to known butyrate producers (Louis and Flint, 2009), which helps to explain the relatively high butyrate formation (24–32% of total SCFA) seen in these fermentors and in previous studies (Mikkelsen *et al*., 2011).

Nutritional specialization with respect to carbohydrate energy sources, particularly among the Firmicutes, is becoming increasingly evident within the human intestinal microbiota. Recent examples among the Ruminococcaceae include the utilization of particulate resistant starch by *Ruminococcus bromii* (Ze *et al*., 2012), of cellulose by *R. champanellensis* (Chassard *et al*., 2011), and of beta‐glucans and xyloglucan by *R. bicirculans* (Wegmann *et al*., 2014). The present study shows that this also applies to members of the Lachnospiraceae and to a substrate as chemically complex as wheat bran. It seems likely that degradation of such substrates requires highly specialized enzymatic machinery and adhesion mechanisms, which have evolved in a relatively small number of species among those that colonize the human large intestine. Enrichment of specific Lachnospiraceae was seen in recent human studies for diets supplemented with whole grain barley (Martinez *et al*., 2013) as well as with wheat bran (Salonen *et al*., 2014). Inter‐individual variation in the abundance of such nutritionally specialized bacteria could affect rates of fermentation of particular plant‐derived fibres and non‐digestible carbohydrates in the large intestine (Walker *et al*., 2011; Ze *et al*., 2013).

The similarity between the particle‐associated and liquid phase microbial communities in the fermentor system is in apparent contrast with the differences between these communities reported previously for human faecal samples (Walker *et al*., 2008). It seems likely, however, that the strong selection imposed here by having wheat bran as the sole energy source in the fermentor system results in species enrichment primarily within the bran‐associated community, with release of cells into the liquid phase reflecting enrichment of the same species. The situation in the distal colon differs in that the diversity of available energy sources is expected to be far greater *in vivo*, with different energy sources likely to be preferentially available to the liquid and particulate phase communities.

Degradation of wheat bran by these fermentor communities resulted in the release of ferulic acid, which was then rapidly hydrogenated in all four experiments to produce PPA derivatives. The reaction sequence for transformation of ferulic acid by the gut microbiota proposed previously (Russell *et al*., 2011) involves hydrogenation to 3‐OMe4‐OHPPA followed by demethylation to give 3,4‐diOHPPA and dehydroxylation to yield 3‐OHPPA (Fig. S3). The four sources of faecal bacteria, however, gave different relative and absolute yields of these PPA derivatives, indicating that there were different patterns of demethylation and dehydroxylation activity between the four faecal microbiotas. Similarly, a recent study showed that the transformation of the phenolic compound chlorogenic acid to form 3OHPPA and PAA was mediated by the gut microbiota, but with considerable inter‐individual variation in the yields of different metabolites (Tomas‐Barberan *et al*., 2014).

The four cultured species that were identified here as being able to degrade wheat bran all released ferulic acid, while strains that did not cause weight loss in wheat bran failed to release ferulic acid. Presumably this is explained by the fact that extensive degradation of the matrix components in plant cell walls, especially arabinoxylan to which ferulate residues are connected by ester linkages, is required for the release of bound ferulic acid from plant cell wall polymers (Akin *et al*., 1993; Iiyama *et al*., 1994; Faulds, 2010). Degradation of highly polymerized core lignins, which are almost entirely composed of phenolic compounds, is considered unlikely under anaerobic conditions (Davin *et al*., 2008). Interestingly, there was limited further conversion of the released ferulic acid in these monocultures, although, as noted above, most of the ferulic acid was found to be transformed into PPA derivatives in the mixed fermentor communities. This suggests that hydrogenation, demethylation and dehydroxylation are largely carried out not by the primary degraders of wheat bran, but by other species within the intestinal microbiota. The formation of ferulic acid‐derived metabolites such as 3‐OHPPA in the human colon is therefore the consequence of a conversion pathway that involves multiple bacterial species. The potential for variation in the metabolome between individuals due to differences in the predominant bacterial species present is therefore considerable. Thus the intestinal, and hence also the systemic, metabolome is not simply determined by dietary intake, but is influenced by an individual's gut microbiota composition. This has the important implication that individuals may benefit from fibre‐rich diets to differing extents depending on their gut microbiota composition.

In conclusion, we show here that particular bacterial species, mainly butyrate‐producing Firmicutes, became strongly enriched from the gut microbiota of four healthy human volunteers during growth on pancreatin pretreated wheat bran under pH‐controlled anaerobic conditions *in vitro*. The fact that this enrichment occurred over time periods (24–48 h) that are within typical colonic transit times suggests that a similar succession is likely to occur *in vivo* following exposure of diet‐derived wheat bran to the complex microbiota of the human proximal colon. The promotion of particular butyrate‐producing bacteria within a diverse microbial community, together with the release of bioactive phenolic compounds during wheat bran degradation, can potentially explain many of the health benefits that are attributed to cereal fibres.

## Experimental procedures

### Bacterial strains

The Firmicutes strains studied here were from stocks held by the authors (S.H. Duncan, Rowett Institute of Nutrition and Health, Aberdeen, UK). *Roseburia intestinalis* L1‐82^T^ (DSM 14610), *Roseburia faecis* M72/1^T^ (DSM 16840), *Roseburia inulinivorans* A2‐194^T^ (DSM 16841), *Eubacterium rectale* A1‐86 (DSM 17629) and *Faecalibacterium prausnitzii* A2‐165 (DSM 17677) (Barcenilla *et al*., 2000; Aminov *et al*., 2006) have also been deposited with Deutsche Sammlung von Mikroorganismen und Zellkulturen (DSMZ). *Ruminococcus bicirculans* 80/3 was isolated as described previously (Wegmann *et al*., 2014). *Butyrivibrio fibrisolvens* 16/4 was isolated as a butyrate‐producing wheat bran degrader (Rumney *et al*., 1995). *Eubacterium siraeum* V10sc8a was isolated from a faecal sample from a young adult female consuming a vegetarian diet. Tenfold serial dilutions of a faecal slurry (10%) in anaerobic M2 medium (pH 6.8) were used to inoculate roll tubes of M2 medium with 0.5% cellobiose as the sole added carbon source (Miyazaki *et al*., 1997) and incubated at 37°C for 48 h. Single colony isolates were re‐purified following a second passage, and one of these isolates was identified as *E. siraeum* from 16S rRNA gene sequencing. The Gram‐negative strain *Bacteroides ovatus* V975 was kindly provided by T.R Whitehead (USDA, Peoria, IL, USA).

### Composition and pretreatment of wheat bran

Food quality wheat bran was obtained from a local supermarket. This material was boiled in dH_2_O and extracted with ethanol under reflux to remove soluble sugars, before being incubated with pancreatin to remove starch (Leitch *et al*., 2007). Chemical analysis showed a total sugar composition of 44.7% xylose, 20.5% arabinose, 27.3% glucose, 2.4% rhamnose, 0.8% mannose, 2.2% galactose and 4.1% uronic acid. The non‐starch polysaccharide (NSP) fraction contained 47.4% xylose, 40.9% arabinose and 8.4% glucose, close to the values reported previously for wheat bran NSP by Edwards and colleagues (2003).

### Degradation of wheat bran by isolated gut bacteria

Pretreated wheat bran (25 ± 1 mg per tube) was added to preweighed culture tubes in triplicate followed by the addition of 7.5 ml M2 basal medium (containing no other carbohydrate energy source) and autoclaving. Tubes were inoculated with 100 μl of an overnight culture of each bacterial strain grown in M2GSC medium (Miyazaki *et al*., 1997). Following incubation for 72 h at 37°C, the tubes were vortex mixed and the bran (but not unattached bacteria) collected at the bottom of the tube by centrifugation at 500 × *g* for 5 min. The bran was then washed four times in sterile distilled water and the bran pelleted each time by centrifugation at 2500 × *g* for 10 min. Residual water was removed and the bran residue was then freeze‐dried. The dried samples were held in a desiccator until tubes with washed residues were re‐weighed and weight losses calculated.

### Model fermentor system

A fermentor system was used to study the ability of mixed faecal microbiota to degrade wheat bran fibre. Glass culture vessels were sterilized by autoclaving at 121°C for 15 min and then connected via pumps to reservoirs containing a supply of fresh, sterile growth medium. The growth medium was based on Hillman and colleagues (1994) (i.e. the medium of Macfarlane *et al*., 1989, with added trace elements), but was modified here to contain a final peptide content of 0.2% and no added carbohydrates. Pretreated wheat bran (3 g) was placed within nylon mesh bags (pore size 5 μm), autoclaved and then submerged in 250 ml fermentor medium to which an SCFA mixture had been added to give 33 mM acetate, 9 mM propionate and 1 mM each of *iso*‐butyrate, *iso*‐valerate and valerate (as in Leitch *et al*., 2007). The purpose of this was to allow growth of any SCFA‐dependent species following inoculation. Growth medium without added SCFA was then pumped continuously into the vessels, which were mixed by a magnetic stirrer bar, at a dilution rate of 0.042 h^−1^, equating to one turnover of 250 ml of medium per day. Inocula were introduced into the nylon bag, which was suspended in the growth medium within the fermentor vessel. Inocula were prepared by mixing 2 g of freshly voided faeces with 8 ml of anaerobic phosphate buffer containing 0.05% cysteine under a CO_2_ atmosphere and vortex mixing. pH was maintained at 6.5 by a pH control unit via addition of sterile 0.5 M HCl or 0.5 M NaOH in response to readings from an internal pH electrode (Broadley James, Electrolab, UK). The temperature was monitored by an internal temperature probe (Electrolab) and was maintained at 37°C by a thermal heating jacket. The vessels were supplied with 100% CO_2_ to the headspace to maintain anaerobic conditions. Fresh faecal samples were provided by four volunteers: one male consuming a vegetarian diet (Donor 1), two females consuming a Western diet (Donors 2 and 3) and a male consuming a Western diet (Donor 4). The volunteers did not take any antibiotics or other drugs known to influence the faecal microbiota for 6 months before the study commenced.

Samples (5 ml) were removed from the nylon bag using a wide bore sterile 10 ml pipette following mixing into a sterile tube. Samples for microbiota analysis were recovered at 2 h, 4 h, 8 h, 24 h and 48 h post‐inoculation. Samples recovered at 0 h and 72 h were also used for metabolite analysis. Each sample was centrifuged at 500 × *g* for 5 min to pellet the wheat bran, and the supernatant was retained as the liquid (L) fraction. The pelleted wheat bran was then washed twice in 5 ml phosphate buffer (pH 6.5), and the bran residue re‐suspended in 5 ml of buffer prior to further manipulation. This is henceforth referred to as the solid (S) fraction. The S samples were placed in a sterile bottle and macerated using a sealed unit homogenizer (Silverson Machines, Bucks, UK). Aliquots for DNA extraction were processed immediately, while those for metabolite analysis were frozen at −80^o^C. Additional aliquots were prepared for FISH analysis as described previously (Walker *et al*., 2008). Four hundred fifty microlitres of 4% paraformaldehyde was added to 50 μg of the S fraction and samples left to fix at 4°C overnight before being applied to gelatin‐coated slides. Dried samples were then hybridized with either the Eub338 or Erec482 probe to assess colonization of the fibres by all bacteria or Lachnospiraceae respectively (Walker *et al*., 2008). Fluorescent cells were visualized with a Leica DMRXA epifluorescence microscope.

### Liquid chromatography‐mass spectroscopy (LC‐MS) analysis

Samples were thawed to 4°C and 80 μl was transferred to an Eppendorf tube. Internal standards (^13^C‐benzoic acid; 80 μl), (2‐amino‐3,4,7,8‐tetramethylimidazo[4,5‐f]quinoxaline; 80 μl) and methanol (160 μl) were then added, and all the reagents were vortex mixed and centrifuged at 12 500 × *g* for 5 min at 4°C). Liquid chromatography separation was performed on an Agilent 1100 HPLC system (Agilent Technologies, Wokingham, UK) using a Zorbax Eclipse 5 μm, 150 mm × 4 mm column (Agilent Technologies). Three different gradients were used to separate the different categories of metabolites, and the mobile phase solvents in each case were water containing 0.1% acetic acid and acetonitrile containing 0.1% acetic acid. In all cases the flow rate was 300 μl min^−1^. The injection volume was 5 μl. The LC eluent was directed into, without splitting, an ABI 3200 triple quadrupole mass spectrometer (Applied Biosystems, Warrington, UK) fitted with a Turbo Ion Spray™ source. For LC methods 1 and 2, the mass spectrometer was run in negative ion mode with the following source settings: ion spray voltage −4500 V, source temperature 400°C, Gases 1 and 2 set at 15 and 40, respectively, and the curtain gas set to 10. For LC method 3, the mass spectrometer was run in positive ion mode with the following source settings: ion spray voltage 5500, source temperature 400°C, Gases 1 and 2 set at 14 and 40, respectively, and the curtain gas set at 10. All the metabolites were quantified using multiple reaction monitoring. Standard solutions (10 ng μl^−1^) for all analytes were prepared and pumped directly via a syringe pump. The ion transitions for each of the analytes were determined based upon their molecular ion and a strong fragment ion. Voltage parameters, de‐clustering potential, collision energy and cell entrance/exit potentials were optimized individually for each analyte.

### SCFA analysis

Short‐chain fatty acids were analysed by gas chromatography following conversion to tert‐butyldimethylsilyl derivatives using a previously published method (Richardson *et al*., 1989), using a mixture of standard purified acids (Sigma Aldrich, Dorset, UK) as references for peak identification. The lower limit for detection of each product was set at 0.2 mM. Dilution of acetate and propionate from the initial medium was calculated from C_t_ = C_t0_.e^−kt^, where C_t0_ is the initial concentration, C_t_ is the concentration (mM) at time t (h), and k is the dilution rate (0.042 h^−1^).

### DNA extractions from fermentor samples

For each collected fermentor sample, 500 μl was placed in a FastDNA SPIN kit for soil (MP Biomedicals, Santa Ana, USA) lysing matrix E tube, and 978 μl of phosphate buffer and 122 μl MT buffer were added to each tube, which were then homogenized in a FastPrep®‐24 instrument following the manufacturer's instructions (MP Biomedicals). The DNA was eluted in 100 μl FastDNA elution buffer.

### 16S rRNA gene sequencing

The DNA prepared from the fermentor samples (S and L fractions) was quantified by Nanodrop and diluted to 25 ng μl^−1^. Variable regions 3–5 of 16S rRNA genes were polymerase chain reaction‐amplified from these DNA extractions following the protocol described by Cooper and colleagues (2013). A GS FLX Titanium 454 (Roche Diagnostics, Oakland) machine was used for sequencing, following the manufacturer's Lib‐L kit protocols. The resulting sequence data are available in the European Nucleotide Archive short‐read archive under study Accession Number ERP005252 and sample Accession Number ERS421604. A list of barcodes used for each sample is shown in Table S2.

### Bioinformatic and statistical analysis

Analysis of the sequence data was carried out using the mothur software package (Schloss *et al*., 2009), as described previously (Cooper *et al*., 2013), except that the minimum sequence length requirement was 200 bases. Following quality control steps, a total of 45 917 sequences remained (median of 1119.5 sequences per sample, range of 54–2808), which formed 406 OTUs at a 97% similarity threshold (Table S1). Data from the solid and liquid phase samples from the 2 h, 4 h, 24 h and 48 h time points from each of the four faecal donors were initially compared using metastats (White *et al*., 2009) analyses in mothur. There were 32 samples included in our comparison between the S and L fractions, and the median Good's coverage for the 16S rRNA gene sequence data from the individual S and L samples was 97.9% (mean 95.8%). metastats‐based comparison of all 406 OTUs detected in the sequence dataset revealed that there were no significant differences between samples recovered from the solid and liquid phase at each time point. As a result, pooled liquid/solid phase data for each donor from each time point were then compared using the LEfSe (Segata *et al*., 2011) software package, as implemented within mothur. Diversity metrics, such as observed OTU diversity, Chao estimates of total diversity, and Shannon and Simpson diversity indices, were calculated in mothur after first subsampling the pooled 4, 24 and 48 h datasets to 1502 reads to ensure equal sampling depth for these comparisons. The Good's coverage estimate at this sequencing depth was greater than 96% for all samples (median: 98.1%). The Kruskal–Wallis test as implemented in minitab (v16) was used to assess whether or not there were differences in diversity between incubation time points and between individual faecal donors. Diversity data were plotted using boxplotr (Spitzer *et al*., 2014). Community structures were compared between faecal donors and between incubation time points using the Bray–Curtis calculator and the dendrogram clustering (tree.shared), PCoA, and AMOVA commands as implemented in mothur.

## Supporting information


**Fig. S1.** Microbial colonization of wheat bran fibres following inoculation by mixed faecal microbiota. Images of wheat bran fibres following incubation with faecal inocula from two donors over time (h) using the FISH probes that detect all bacteria (Eub338) or Lachnospiraceae (Erec482).

**Fig. S2.** Impact of inter‐individual variation, and enrichment with wheat bran, on microbial community structure. Dendrogram, generated using the Bray–Curtis calculator in mothur, showing dissimilarities between microbial communities from each of the four donors at each time point, and the nine enriched Lachnospiraceae OTUs plus *Bacteroides uniformis* for the four donors and three time points (16S rRNA gene sequence data shown in Table S1 and in Figs 1–3 in the main text).

**Fig. S3.** Steps in ferulic acid conversion by the faecal microbiota. The major metabolites of ferulic acid detected in faecal samples (Russell *et al*., 2011) and in the fermentor experiments described in Fig. 4 are shown, together with reactions involved in their conversion. Abbreviations are given below the name of each compound.

**Table S1.** Time sequence of bacterial colonization of wheat bran, from 454 sequence analysis of amplified 16S rRNA genes for four fermentor communities inoculated with human faecal microbiota. Table shows the proportional abundance and taxonomic classification for each OTU, and the LEfSe analysis (calculated using pooled solid and liquid sample data) comparing proportional abundance of each OTU at the 4 h and 48 h time points and between faecal donors.

**Table S2.** Golay barcode sequences that were incorporated into the 16S rRNA gene primers that were used for each sample.
