# Supplementary figures and images for: Wheat bran promotes enrichment within the human colonic microbiota of butyrate‐producing bacteria that release ferulic acid
Source: Environ Microbiol. 2016 Jan 21;18(7):2214–25. doi: 10.1111/1462-2920.13158 (PMC4949515; doi:10.1111/1462-2920.13158)

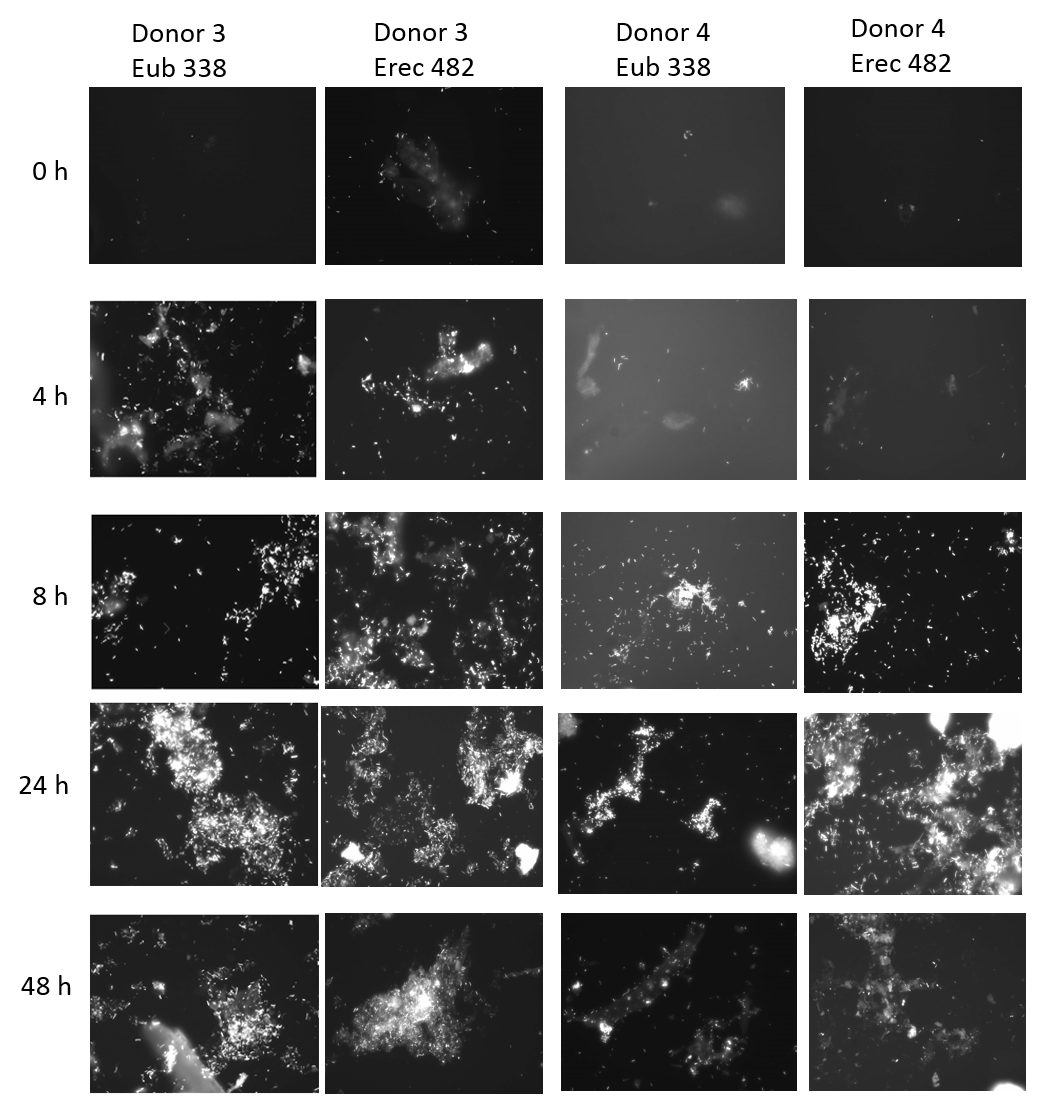

Supplement: Supplementary file 1 — Fig. S1. Microbial colonization of wheat bran fibres following inoculation by mixed faecal microbiota. Images of wheat bran fibres following incubation with faecal inocula from two donors over time (h) using the FISH probes that detect all bacteria (Eub338) or Lachnospiraceae (Erec482). [file EMI-18-2214-s001.tif]

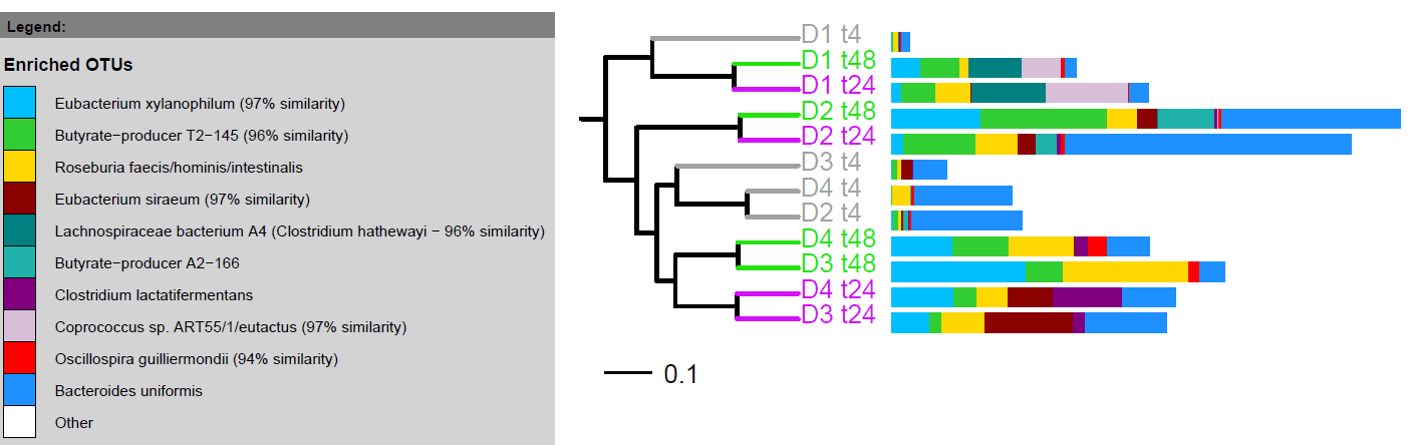

Supplement: Supplementary file 2 — Fig. S2. Impact of inter‐individual variation, and enrichment with wheat bran, on microbial community structure. Dendrogram, generated using the Bray–Curtis calculator in mothur, showing dissimilarities between microbial communities from each of the four donors at each time point, and the nine enriched Lachnospiraceae OTUs plus Bacteroides uniformis for the four donors and three time points (16S rRNA gene sequence data shown in Table S1 and in Figs 1–3 in the main text). [file EMI-18-2214-s002.tif]

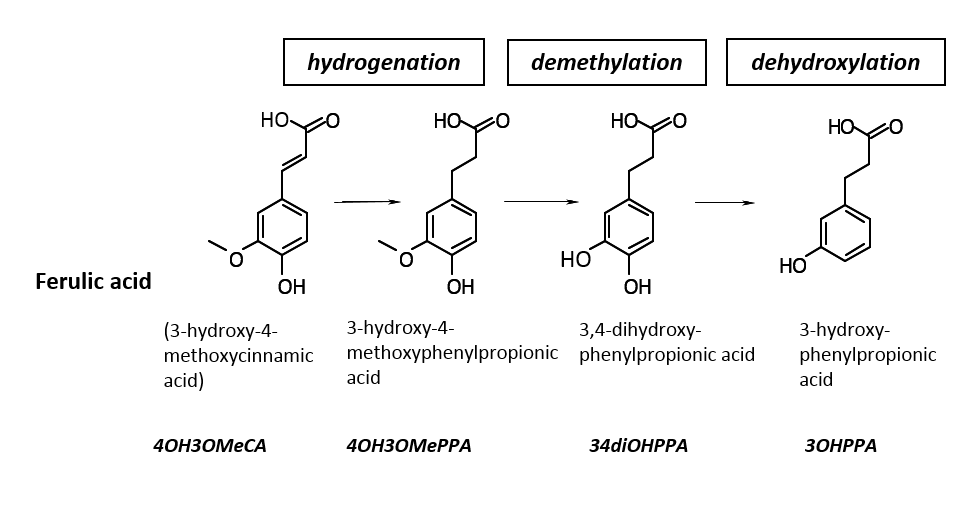

Supplement: Supplementary file 3 — Fig. S3. Steps in ferulic acid conversion by the faecal microbiota. The major metabolites of ferulic acid detected in faecal samples (Russell et al., 2011) and in the fermentor experiments described in Fig. 4 are shown, together with reactions involved in their conversion. Abbreviations are given below the name of each compound. [file EMI-18-2214-s003.tif]
